# Medicolegal analysis of physical violence toward physicians in Egypt

**DOI:** 10.1038/s41598-024-60857-2

**Published:** 2024-05-13

**Authors:** Rasha Ismail Khedr, Omneya Ibrahim Mohamed, Zahraa Khalifa Sobh

**Affiliations:** 1 Forensic Medicine and Clinical Toxicology Department, Faculty of Medicine, Alexandria University, Alexandria, Egypt; 2 Present Address: Faculty of Medicine, Alexandria University, Champollion Street, Alexandria, 5372006 Egypt

**Keywords:** Workplace violence, Physical violence, Physicians, Medicolegal, Egypt, Health care, Health occupations, Medical research

## Abstract

This study analyzed physical violence against physicians in Egypt from a medicolegal perspective. 88%, 42%, and 13.2% of participants were exposed to verbal, physical, and sexual violence. Concerning the tools of violence, 75.2% of attackers used their bodies. Blunt objects (29.5%), sharp instruments (7.6%), and firearm weapons (1.9%) were used. The commonest manners of attacks were pushing/pulling (44.8%), throwing objects (38.1%), and fists (30.5%). Stabbing (4.8%) and slashing (2.9%) with sharp instruments were also reported. Traumas were mainly directed towards upper limbs (43.8%), trunks (40%), and heads (28.6%). Considering immediate effects, simple injuries were reported that included contusions (22.9%), abrasions (16.2%), and cut wounds (1.9%). Serious injuries included firearm injuries (4.8%), internal organs injuries (3.8%), fractures (2.9%), and burns (1.9%). Most (90.5%) of injuries healed completely, whereas 7.6% and 1.9% left scars and residual infirmities, respectively. Only 14.3% of physicians proceeded to legal action. The current study reflects high aggression, which is disproportionate to legal actions taken by physicians. This medicolegal analysis could guide protective measures for healthcare providers in Egypt. In addition, a narrative review of studies from 15 countries pointed to violence against physicians as a worldwide problem that deserves future medicolegal analyses.

## Introduction

Physicians, like any professionals, might be exposed to violence in their workplaces. The World Health Organization (WHO) defined Work Place Violence (WPV) as an intentional use of threat or power against the person(s) in work-related circumstances that results in psychological harm, injury, or even death^1^.

Physicians are highly liable for being victims of the aggression perpetrated by patients or their relatives. Physicians are exposed to much more WPV than other professionals, as reported by the Occupational Safety and Health Administration (OSHA)^2^. Also, the Bureau of Labor Statistics showed that WPV against healthcare workers is four times greater than in other sectors^3^. Violence in healthcare sectors endangers both healthcare providers and patients. Thus, violence against physicians severely impacts the healthcare system as a whole^4^.

The literature published in the last few years pointed to increasing violence against healthcare providers, including physicians, in the Arab world, such as Egypt^5,6^, Saudi Arabia^7^, Jordan^8^, Palestine^9^, and Iraq^10^. In addition to these Middle Eastern countries, literature from Turkey^11^, China^12,13^, Italy^14^, Barbados^15^, Myanmar^16^, India^17^, and the USA^18^ notified aggressive behavior toward medical staff. Recently, violence in healthcare systems gained global attention during the COVID-19 pandemic^19,20^.

It is worth mentioning that most of the studies investigated violence against healthcare providers as a public health problem and discussed this phenomenon from occupational and community medicine perspectives^5,7,13,14,21–23^. Other studies concerned the characterization of violence against healthcare providers during the COVID-19 crisis^20,24,25^. Nevertheless, scarce data are available regarding the medicolegal aspects of physical violence against physicians.

Egypt is the largest country in the Arab world by the size of its population, as it is inhabited by 34.3% of the Arabian people. Egyptian physicians provide healthcare services in a nation with more than 100 million population with a two percent annual growth rate^26,27^. Like other countries, Egyptian studies denoted high violence against healthcare providers^24,25,28^. Adoption of protective measures for healthcare providers should be based upon a comprehensive analysis of violent events in healthcare facilities. Thus, the current study had an in-depth look at the medicolegal aspects of physical violence toward physicians in Egypt.

## Methods

### Study design

The current study is a cross-sectional descriptive study. This questionnaire-based research was carried out on Egyptian physicians practicing medicine in Egypt.

### Sample size calculation

Epi Info-7 program was used to calculate the sample size considering adjusting power at 80%, confidence level 95.0%, and the prevalence of physical violence against physicians is 12.1%^25,29^. The minimum estimated sample size was 163 participants; it was increased to 250 participants to account for nonresponse and to improve the study power.

The following formula was used:

$$\left[ {{\text{S}} = {\text{Z}}^{2} \times {\text{P}} \times (1 - {\text{P}})/{\text{M}}2} \right],$$

where; S = sample size for infinite population, Z = Z score(1.96), P = population proportion (0.121), M = Margin of error (0.5).

### Eligibility criteria

This research was carried out on a convenient collected sample of registered Egyptian physicians who practiced medicine in different Egyptian governorates at the time of the conduction of the study. The study included responses received during March and April 2023. Non-Egyptian physicians and Egyptian physicians who practiced medicine in countries other than Egypt were excluded from the study.

### Piloting

A pilot study encompassing 20 Egyptian physicians was performed before the study to test the clarity of the questions and the approximate time needed to provide complete responses to the questionnaire. The feedback from the piloting proved that the questionnaire was well-formulated. The sample of the pilot study was excluded from the original study sample.

### Data collection tool

The questionnaire was designed to investigate physical violence against physicians in Egypt from a medicolegal perspective, and it was formulated following a comprehensive review of the literature in this context^4,22–25,30^. Google form was used to formulate the survey that was electronically distributed to the participants. We enabled Google Forms’ built-in ‘limit to 1 response to avoid multiple responses by the same participants.

The questionnaire comprised 25 questions that covered six sections, as follows:

#### Personal and professional data (Six questions)

- Personal data: age and gender.
- Professional data: specialty, years of experience, qualification, and job level.

#### Exposure to physical violence, verbal violence, or sexual harassment (One question)

#### Frequency and trend analysis of physical violence (Three questions)

- Year﻿(s) when physical violence occurred.
- Frequency of attacks of physical violence per year.
- Relation between COVID-19 crisis and frequency of physical violence.

#### Analysis of the attacks of physical violence (Eight questions)

- Healthcare institute where physical violence occurred.
- Time of the violence.
- Person(s) who committed the physical violence.
- Personal characters of the person who committed the physical violence.
- Reason for the outbreak of physical violence.
- Instrument used in the physical violence.
- Manner(s) of attack.
- Part(s) of the body attacked during the violence.

#### Consequences of physical violence (Five questions)

- Immediate health effects of physical violence.
- Long-term consequences of the violence.
- Psychological effect of physical violence.
- Reactions of physicians in the attacks of physical violence.
- Legal outcome of the physical violence.

#### Root causes of physical violence and proposed solutions (Two questions)

- Root causes of increasing violence against physicians in Egypt.
- Proposed solutions for protecting physicians against violence in the future.

At the beginning of March 2023, the questionnaire was distributed on medical websites frequently accessed by thousands of physicians in Egypt 'Supplementary data'. In addition, physicians from various Egyptian governorates were contacted and invited to participate in the survey and to disseminate questionnaires among physicians. The objectives of the study were declared. It was mentioned that approximately 10 min were needed to respond to the questionnaire.

### Ethical considerations

Ethical appoval was obtained from the Research Ethics Committee of the Faculty of Medicine, Alexandria University (IRB Number: 00012098, FWA Number: 00018699, Approval serial number: 0304728). The study was in accordance with the ICH-GCP Guidelines and applicable local and institutional regulations and guidelines that govern the ethics Committee's operation. The study's brief description and aim were mentioned at the beginning of the questionnaire. Informed consent was obtained from all study participants. The personal data of the respondents was kept private and confidential.

### Data analysis

Data were analyzed using IBM SPSS software package version 20.0. (Armonk, NY: IBM Corp). The chi-square test was applied to study the association between the categorical variables. Continuous data was tested for normality using the Kolmogorov–Smirnov. The significance was judged at the 5% level.

## Results

The current study included the responses of 250 physicians from 13 Egyptian governorates: Cairo, Alexandria, Giza, El Beheira, Beni Suef, Dakahlia, Menofia, Assiut, Al Qalyubia, Sohag, Gharbia, Kafr El Sheikh, and Sharqia.

Table 1 demonstrates that 86.4% of participants aged less than 40 years, with a mean age of 35.0 ± 5.51 years. More than two-thirds (68.8%) of respondents had experienced less than ten years, with a mean of 8.17 ± 5.19 years. More than half (56%) of the participants had a Master’s degree, 34.8% had a bachelor’s degree, and the rest had a doctorate. The specialists and residents constituted 51.2% and 39.2% of the participants. Internal medicine physicians, surgeons, and emergency physicians represented 38.8%, 21.2%, and 20% of participants, respectively.

**Table 1:** Relation between participants’ data and exposure to physical violence (n = 250).

Personal and professional data	Total	Exposure to physical violence				χ^2^	P
	n (%)	Yes (n = 105)		No (n = 145)			
		No	%	No	%		
Age							
25–< 30 years	51 (20.4)	15	29.4	36	70.6	4.189	0.123
30–< 40 years	165 (66)	75	45.5	90	54.5		
> 40 years	34 (13.6)	15	44.1	19	55.9		
Gender							
Male	48 (19.2)	33	68.8	15	31.3	17.450	< 0.001*
Female	202 (80.8)	72	35.6	130	64.4		
Years of experience							
< 5 years	81 (32.4)	28	34.6	53	65.4	10.279	0.036*
5–< 10 years	91 (36.4)	38	41.8	53	58.2		
10–< 15 years	52 (20.8)	25	48.1	27	51.9		
15–< 20 years	19 (7.6)	13	68.4	6	31.6		
> 20 years	7 (2.8)	1	14.3	6	85.7		
Qualification							
Bachelor	87 (34.8)	34	39.1	53	60.9	0.682	0.711
Master degree	140 (56)	62	44.3	78	55.7		
Doctorate	23 (9.2)	9	39.1	14	60.9		
Job level							
Resident	98 (39.2)	38	38.8	60	61.2	2.039	0.361
Specialist	128 (51.2)	59	46.1	69	53.9		
Consultant	24 (9.6)	8	33.3	16	66.7		
Specialty							
Internal medicine	97 (38.8)	36	37.1	61	62.9	12.598	0.013*
Surgery	53 (21.2)	30	56.6	23	43.4		
Emergency	50 (20)	28	56.0	22	44.0		
Pediatrics	42 (16.8)	15	35.7	27	64.3		
Others	8 (3.2)	1	12.5	7	87.5		

χ^2^: Chi square test.

p: p value for comparing between the studied categories.

*Statistically significant at p ≤ 0.05.

**Figure 1:**
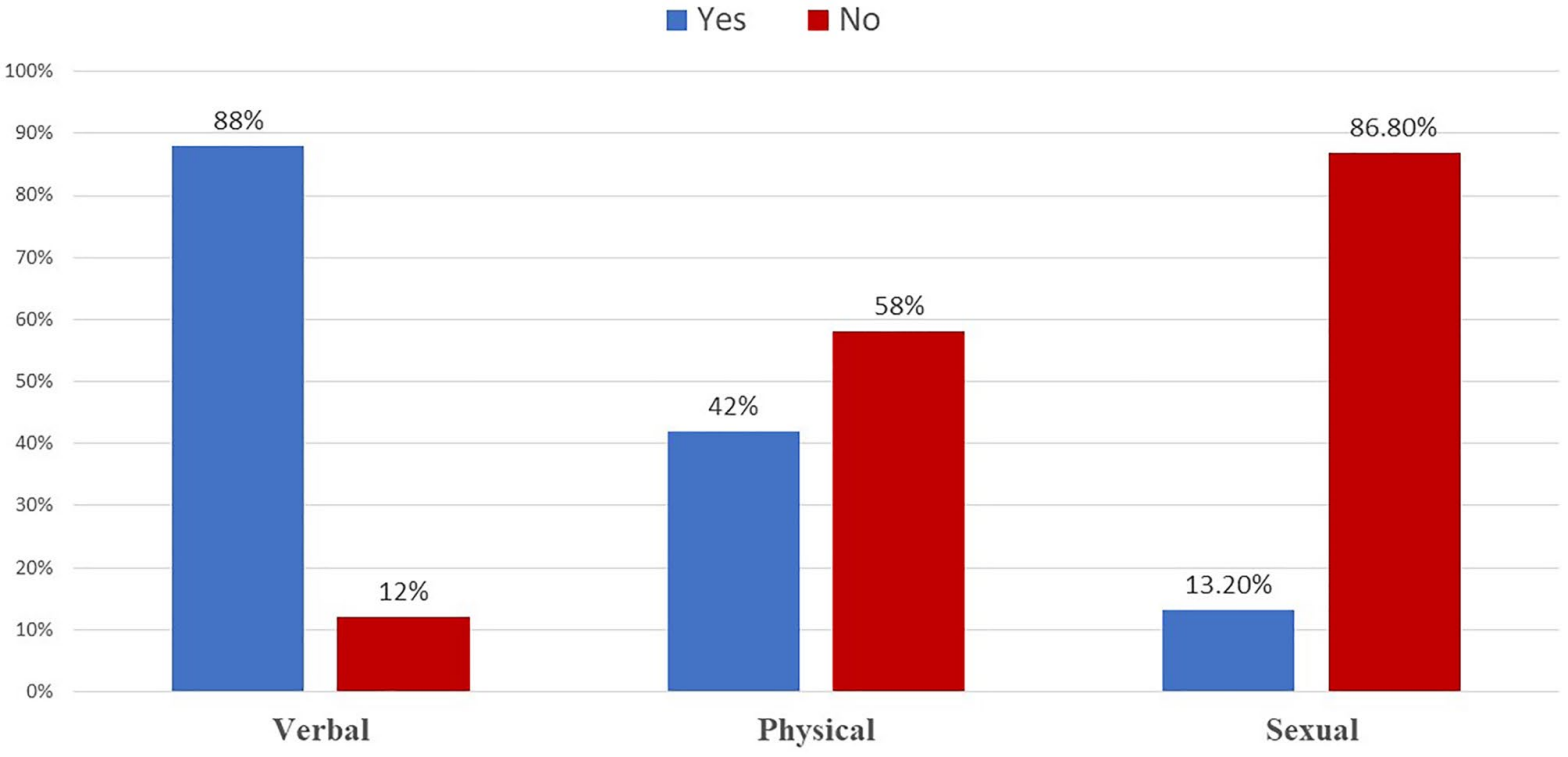
Type of violence experienced by the participating physicians (n = 250).

Figure 1 elucidates that 88% of participants had been exposed to verbal violence, whereas 42% were subjected to physical violence. Only 13.2% of participants were exposed to sexual harassment. There was a significant association between exposure to physical violence and verbal violence (χ^2^ = 20.924, *p*-value < 0.001), whereas there was no significant association between exposure to physical violence and sexual harassment (χ^2^ = 2.456, *p*-value = 0.117) 'Supplementary Table 1'.

### Relation between participants’ data and exposure to physical violence

Table 1 shows that more than two-thirds (68.8%) of the physicians exposed to physical violence were males, and there was a statistically significant association between the male gender and exposure to violence (χ^2^ = 17.450, p =  < 0.001).

Regarding the year of experience, physical violence was significantly affected by the year of experience (t = 10.279, p = 0.036). It was observed that more than two-thirds (68.4%) of the physicians with experience duration of 15- 20 years were exposed to physical violence.

Also, the exposure to physical violence was significantly affected by the physicians’ specialties (t = 12.598, p = 0.013). Specialties in which the numbers of physicians exposed to physical violence outnumbered non-exposed physicians were surgery and emergency medicine.

There was no statistically significant association between exposure to physical violence and age (p = 0.123), qualification (p = 0.711), or job levels (p = 0.361).

### Frequency and trend analysis of the physical violence

**Figure 2:**
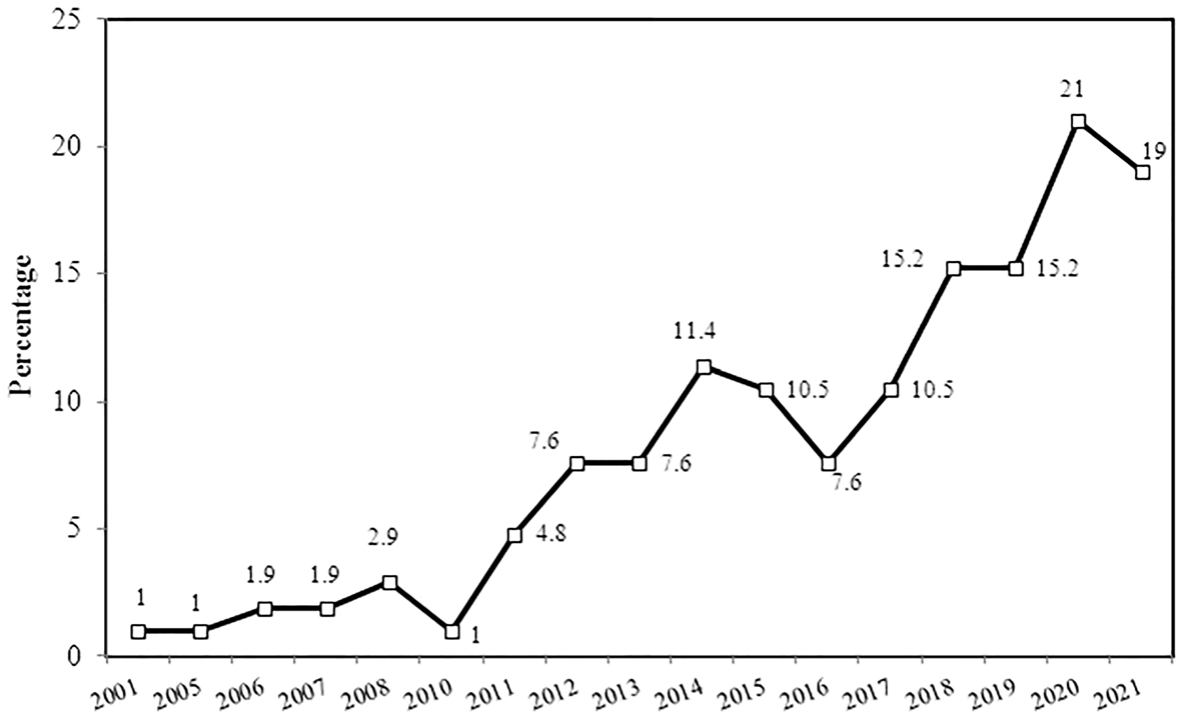
Trend of reported attacks of physical violence in Egypt (n = 105).

In the current study, 30.5% of the respondents had been exposed to physical violence more than five times per year, and 28.6% of the physicians were exposed to physical violence once yearly. A great majority (87.6%) of the physicians exposed to physical violence denoted a rising trend of physical violence in Egypt. However, 61.9% of the physicians exposed to physical violence reported no relation between the COVID-19 crisis and increased violence 'Supplementary Table 2'. The reported physical violence trend analysis elucidated a significant rise in violent attacks following 2011. Forty percent of episodes of physical violence have been reported since 2020 (Fig. 2).

### Analysis of the attacks of physical violence

Table 2 shows that Ministry of Health hospitals are the most common institute where the participants experienced physical violence (60%), followed by university hospitals (42.9%). Physical violence attacks were more reported in the night shifts (67.6%).

**Table 2:** Analysis of physical violence according to responses of participating physicians exposed to physical violence (n = 105).

Physical violence	No	%
What is the heath care institute where you experienced physical violence took place?*		
Ministry of health hospital	63	60.0
Univerisity hospital	45	42.9
Private hospital, Clinic	26	24.8
When did this physical violence take place? *		
In the morning shift (8 a.m. to 2 p.m.)	34	32.4
In the evening shift (2 p.m.to 8 a.m.)	44	41.9
In the night shift (8 p.m.to 8 a.m.)	71	67.6
Who commits the violent attack?		
Relative(s), friend(s) of the patient(s)	89	84.8
Patient himself	12	11.4
What was/were character(s) related to person(s) who commit the physical violence?*		
Low education standard	78	74.3
Poor communication skills	45	42.9
Drug abuse	31	29.5
Psychiatric disorders	11	10.5
What was the reason for the outbreak of physical violence? *		
Shortage of equipment and supplies necessary for management	52	49.5
Stress and over concern about patient condition	48	45.7
Long waiting for receiving medical care	45	42.9
Delivering of bad news	27	25.7
No apparent reason	24	22.9
Unexpected death of the patient	23	21.9
Occurrence of complication(s)	18	17.1
High fees for healthcare service	10	9.5
Medical error result in patient harm (malpractice)	3	2.9
What is the instrument(s) used in the physical violence?*		
Body of attacked person (hand, head, leg)	79	75.2
A blunt object (as chair, wooden stick)	31	29.5
A sharp instrument (as knife)	8	7.6
Firearm weapon (such as gun)	2	1.9
What is the manner(s) of attack?*		
Pushing or pulling of the physician	47	44.8
Throwing of an object	40	38.1
Blows from a hand (fist)	32	30.5
Kicking by leg or foot	25	23.8
Slabbing by hand	16	15.2
Striking with a blunt object(s)	15	14.3
Attempt of stabbing with sharp object(s)	5	4.8
Slashing (cutting) by sharp object(s)	3	2.9
What is /are part(s) of your body attacked during violence?*		
Upper limb(s)	46	43.8
Chest & Abdomen	42	40.0
Head	30	28.6
Back	15	14.3
Lower limb(s)	15	14.3

*More than one answer could be selected.

The great majority (84.8%) of persons who committed violence toward physicians were patients’ relatives and friends. 74.3% and 42.9% of physicians mentioned low education standards and poor communication as characteristics of attackers. The most commonly reported causes of violence were a shortage of medical equipment and supplies (49.5%), stress conditions (45.7%), and long waiting times (42.9%).

Concerning the instruments used in violence, three-quarters (75.2%) of the participants reported that the attackers used their bodies. Blunt objects were mentioned by 29.5% of participants as violent tools, whereas 7.6% and 1.9% of the physicians were attacked by sharp objects and firearm weapons, respectively.

Regarding the manners of attacks, 44.8%, 38.1%, and 30.5% of the physicians were subjected to pushing/pulling, throwing objects on them, and hand fists, respectively. Attempts of stabbing (4.8%) and slashing (2.9%) with sharp instruments were also mentioned. The traumas were mainly directed toward upper limbs, trunks, and heads, as described by 43.8%, 40%, and 28.6% of physicians, respectively.

### Consequences of the physical violence

Table 3 elucidates that three-quarters (75.2%) of the physicians experienced pain due to exposure to violence. Simple wounds were mentioned that included contusions (22.9%), abrasions (16.2%), and cut wounds (1.9%). Also, serious injuries were reported that included firearm injuries (4.8%), internal organs injuries (3.8%), fractures (2.9%), and burns (1.9%). Most (90.5%) violence-induced injuries healed completely, whereas 7.6 left scars. Most seriously, 1.9% of the physicians reported residual infirmities due to violence.

**Table 3:** Consequences of the physical violence according to responses of participating physicians exposed to physical violence (n = 105).

Consequences of physical violence	No	%
What was/were the immediate result(s) of physical violence?*		
Pain	79	75.2
Contusion	24	22.9
Abrasion	17	16.2
Bleeding	8	7.6
Contused wound	5	4.8
Firearm injury	5	4.8
Serious internal organs injuries	4	3.8
Fracture	3	2.9
Cut wound	2	1.9
Burn	2	1.9
What were the long-term consequences of the violence?		
Complete recovery	95	90.5
Healed with skin scar	8	7.6
Infirmity (residual disability)	2	1.9
What Was/were effect(s) of physical violence on your psychological status?*		
Anger	62	59.0
Depression	54	51.4
Anxiety	41	39.0
Fear	21	20.0
Post traumatic stress disorder (PTSD)	15	14.3
How did you react to the act of physical violence towards you?*		
Asked the security personnel for help	62	59.0
Verbal response	45	42.9
Called the police	36	34.3
Left the place	27	25.7
Reported to the manager	27	25.7
Physical response	23	21.9
Asked colleagues for help	21	20.0
Did nothing	13	12.4
What was the legal outcome of the violence?		
Nothing happened	57	54.3
Attacking person(s) made an excuse and you forgive him	33	31.4
Legal and judicial actions	15	14.3

*More than one answer could be selected.

The psychological impact of the violence included anger (59%), depression (51.4%), anxiety (39%), fear (20%), and posttraumatic stress disorder (14.3%). The most common responses of physicians following exposure to violent incidents were asking the security personnel for help (59%), verbal response (42.9%), and calling the police (34.3%).

Regarding the legal outcome, more than half (54.3%) of physicians reported that no legal actions had been taken to respond to the violence. 31.4% of physicians accepted the assailants’ excuses. Only 14.3% of the physicians proceeded to legal and judicial actions.

### Root causes of violence and proposed solutions

**Figure 3:**
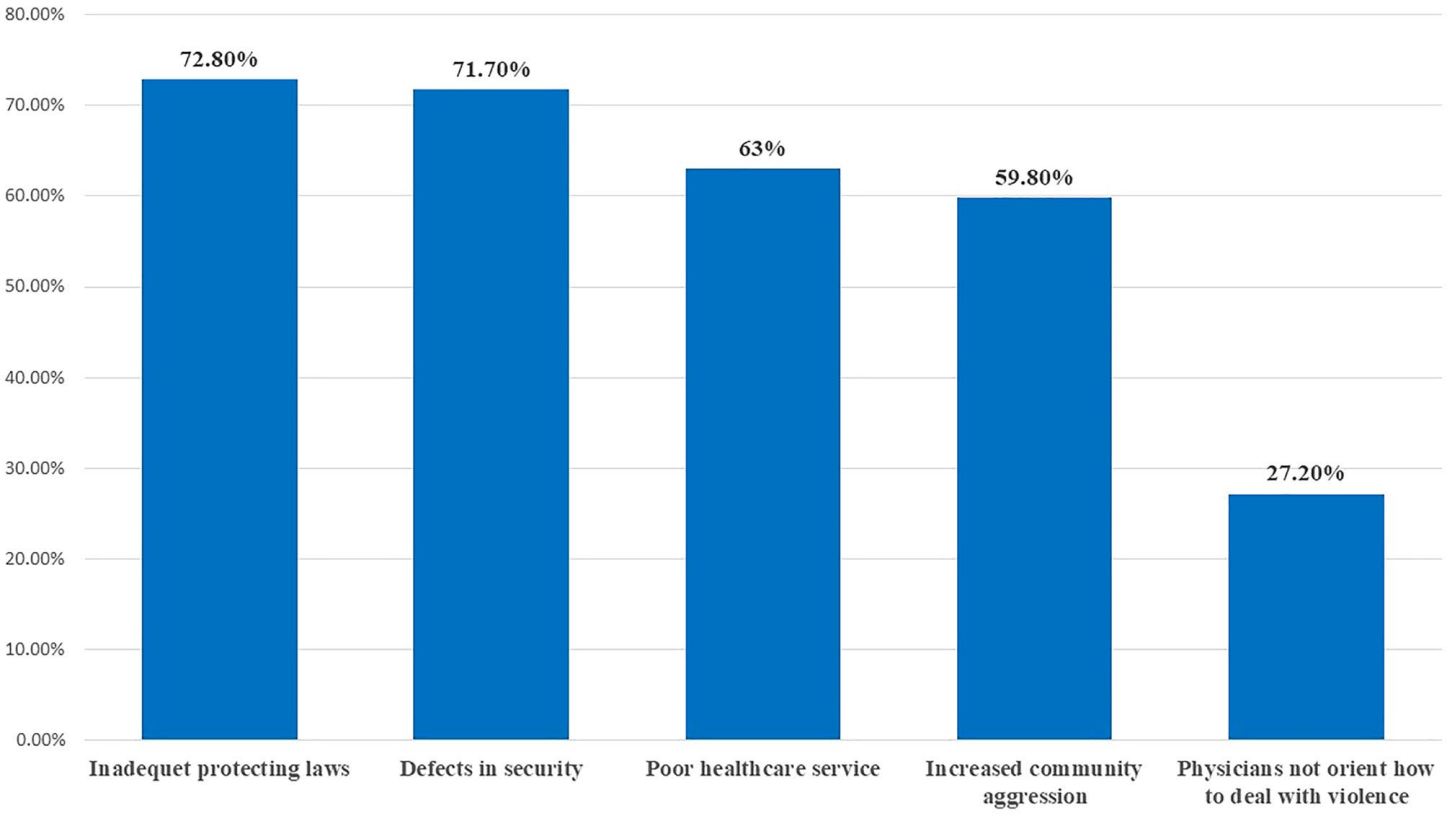
Root causes of increasing physical violence in Egypt according to responses of physicians previously exposed to physical violence (n = 105).

Increased violence in Egypt was attributed to inadequate laws that protect healthcare providers (72.8%), defects in security in healthcare facilities (71.7%), and poor healthcare services (63%), as illustrated in Fig. 3.

**Figure 4:**
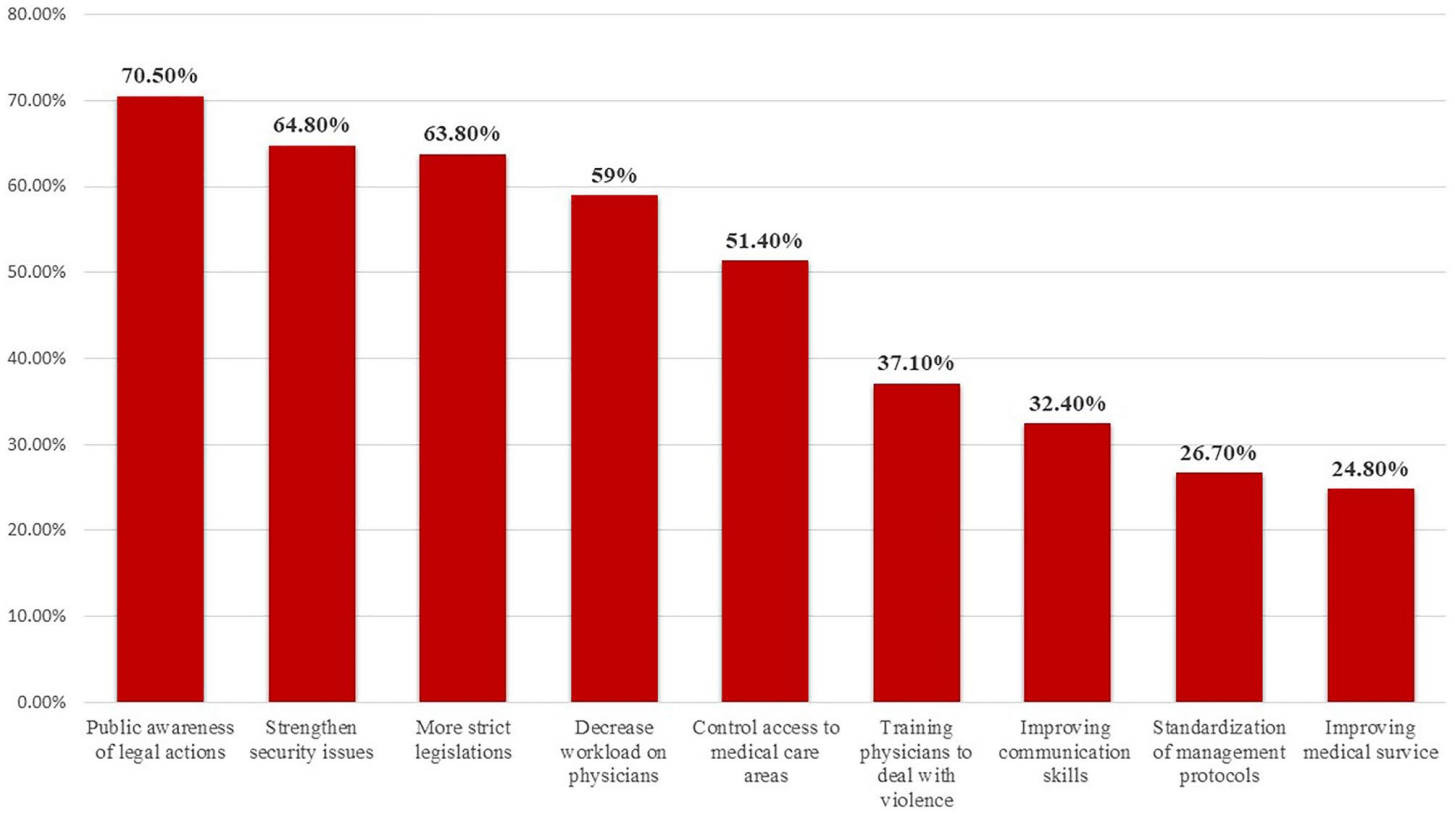
Solutions proposed by the physicians previously exposed to physical violence (n = 105) to protect physicians from violence.

Physicians exposed to physical violence proposed solutions to protect physicians in the future that included increasing the public awareness of the legal consequence of violence against physicians (70.5%), strengthening security issues (64.8%), adopting strict legal actions to protect healthcare providers (63.8%) and decreasing the workload by increasing number of healthcare providers (59%), as shown in Fig. 4.

## Discussion

Violence against medical staff is a deep-rooted problem in different countries. The present study adopted a self-administered web-based questionnaire to analyze medicolegal aspects of violence toward Egyptian physicians. Over three-quarters of the participants were under 40, and more than two-thirds practiced medicine for ten years or less. The participation of a high percentage of new generations could be attributed to their accessibility to online webpages through which the questionnaire was distributed more than seniors^31,32^.

The majority (88%) of participants reported previous exposure to verbal violence, whereas 44% were exposed to physical violence, and there was a significant association between the occurrence of verbal and physical violence. On the other hand, only 13% of respondents reported exposure to sexual harassment that was not significantly related to physical violence.

A narrative review included published questionnaire-based studies that investigated violence in healthcare sectors since 2000. Table 4 illustrates 25 studies from 15 countries other than the present study, pointing to violence in healthcare institutes as a worldwide problem. The percentages of healthcare providers who reported verbal violence ranged from 8.7 to 94.6%. Whereas the percentages of those exposed to physical violence ranged from 1 to 81%. It was found that verbal violence was more prevailing in relation to physical violence in all studies. Regarding sexual violence, it was investigated by only five studies, other than the present study^9,21,28,33,34^. The percentages of medical staff who reported sexual harassment ranged from 1.7 to 48.1%.

**Table 4:** Studies investigated violence against healthcare providers since 2000.

Study	Year	Participants number	Category of healthcare providers	Country	Type of violence			Affiliation of authors/journal scope
					Verbal	Physical	Sexual	
Ness et al.^35^	2000	380	Physicians	United Kingdom	54.0%	6.0%	–	Psychiatry
Forrest et al.^36^	2011	804	Physicians	Australia	58%	18%	–	Primary healthcare
Talas et al.^33^	2011	270	All staff in emergency department	Turkey	79.6%	41.1%	15.9%	Health science
Algwaiz and Alghanim^7^	2012	383	physicians and nurses	Saudi	94.6%	12%	–	Public Health
Elkhawaga et al.^21^	2012	68	Emergency Physicians	Egypt	76.5%	60.3%	30.9%	Public Health
Kitaneh and Hamdan^9^	2012	240	physicians and nurses	Palestine	38.3%	20.8%	1.7%	Public health
Abdel-Salam^5^	2014	335	Emergency Physicians	Egypt	78.6%	21.4%	–	Public Health
Da Silva et al.^37^	2015	2940	physicians ,nurses ,Security	Brazil	52.5%	23%	–	Public Health
Erdur et al.^11^	2015	174	Emergency Physicians	Turkey	43%	5.2%	–	Emergency Medicine
Shi et al.^12^	2015	1,656	Physicians	China	92.8%	81%	–	Public Health
Terzoni et al.^14^	2015	903	All hospital workers	Italy	40.2%	11.5%	–	Public Health
Xing et al.^13^	2015	840	physicians and Nurses	China	–	12.6%	–	Public Health
Abed et al.^15^	2016	102	physicians ,nurses	Barbados	60%	3%	–	Public Health
Abdellah and Salama^6^	2017	134	emergency physicians, nurses	Egypt	58.2%	15.7%	–	Public Health/Emergency medicine
Kasai et al.^16^	2018	196	Physicians	Myanmar	8.7%	1%	–	Public Health
Zhu et al.^34^	2018	1300	physicians of obstetrics and gynaecology	China	66.7%	18.8%	5%	Public Health
Kumar et al.^17^	2019	118	Critical Care Physicians	India	67%	19%	–	Critical Care Medicine
Sharma et al.^17^	2019	295	physicians and nurses	India	50%	3.7%	–	Critical Care Medicine
Hameed et al.^10^	2020	426	All staff in emergency department	Iraq	33.2%	14.3%	–	Nursing
Alhamad^8^	2021	611	Physicians	Amman	63.5%	10.4%	–	Emergency Medicine
McGuire et al.^18^	2021	242	All staff in the emergency department	USA	86%	37%	–	Emergency Medicine
Mahmoud and Ezzat^23^	2022	445	Physicians	Egypt	71.1%	21.3%	–	Public Health
Assil et al.^28^	2022	108	Emergency physicians and nurses	Egypt	86.1%	34.3%	48.1%	Forensic Medicine
Elsaid et al.^25^	2022	405	Healthcare workers	Egypt	87.9%	1.6%	–	Forensic Medicine
Salem et al.^30^	2022	171	Physicians	Egypt	40.3%	14.2%	–	Forensic Medicine
Current Study	2023	250	Physicians	Egypt	88%	42%	13.2%	Forensic Medicine

It was noticed that the published studies investigated violence in healthcare sectors through perspectives of public health, emergency medicine, critical care, psychiatry, and nursing. Only three out of 25 studies were conducted by researchers affiliated with forensic medicine^25,28,30^; these forensic articles were concerned with violence in the Egyptian healthcare sector during the COVID-19 pandemic.

The current study concerned analyzing medicolegal aspects of violence regardless of the COVID-19 crisis. This study started by outlining the characteristics of physicians previously exposed to physical violence. It was observed that more than two-thirds of them were males, which agreed with the Egyptian study conducted by Mahmoud and Ezzat^23^. In other words, In Egypt, female doctors were significantly less exposed to physical violence than their male counterparts, which could be attributed to the local cultural norms that dignified females' bodies. On the other hand, Assil et al. reported that emergency physicians in Egypt of both sexes were equally exposed to aggression during the COVID-19 crisis^28^. Elsaid et al. declared that Egyptian female healthcare providers experienced more physical violence than males during the COVID-19 pandemic^25^. Variations in the included categories of healthcare providers and the circumstances of the COVID-19 crisis might explain the variations in the results regarding the relationship between gender and physical violence.

This study revealed that physicians aged 30 years were more exposed to physical violence than junior physicians who agreed with Abdel-Salam^5^. Also, it was found that physicians with experience duration from 10 to 20 years were significantly more exposed to physical aggression than others.

It is essential to consider that complicated cases are managed by more senior physicians with relatively long experience duration. Working on risky issues could be associated with undesirable outcomes that provoke anger and attacks of violence toward treating staff^38^. Also, the more extended period of medical practice and handling many cases increase the likelihood of exposure to violent events. On the other hand, a study conducted by Algwaiz and Alghanim in Saudia Arabia found that less experienced healthcare workers were exposed to physical violence^7^. Including nursing staff in the Algwaiz and Alghanim study might explain such variations in results.

In the present work, physical violence was significantly higher in specialties that manage critical cases, including surgery and emergency. The severity and life-threatening conditions of the cases admitted to these departments are often associated with poor outcomes. Anger from the patients ' families could be transformed into violence toward the physicians. Similarly, several studies pointed to emergency departments as a commonplace of violence toward medical staff^8,11,18^.

The current study revealed that physicians' qualifications and job levels did not influence the exposure of physicians to physical violence, which means that increasing scientific degrees or job levels does not protect physicians against physical violence.

Regarding the trend and frequency of violence, the present study pointed to significant increases in physical violence toward physicians in Egypt, particularly after 2011. It is essential to consider that the Arab Spring Revolutions were associated with increased violence and aggressive behavior among Arabian societies in different sectors, including healthcare systems^39^.

It is worth mentioning that nearly two-thirds of participants stated that increased violence toward medical staff was not influenced by the COVID-19 crisis, which coincides with the results of Salem et al.^30^. However, a meta-analysis conducted by Ramzi et al. proved that violence significantly increased against nursing staff, not physicians, during the COVID-19 pandemic^19^.

The present study reported an extremely high frequency of violent incidents, as 30.5% of the physicians had been exposed to physical violence more than five times annually.

Analysis of the circumstances of violence revealed that the most violent events occurred in the hospitals of the Ministry of Health and that of universities. Similarly, Abdellah and Salama; and Mahmoud and Ezzat previously reported that violence toward emergency physicians was more prevailing in Egyptian governmental hospitals^6,23^. The governmental institutes in Egypt serve most populations, particularly those who cannot afford the cost of treatment in private institutes. Also, tertiary governmental hospitals receive emergency and complicated cases that private hospitals cannot manage^31,32^. The high workload could stand behind relative medical supply shortages and waiting times that promote violence toward medical staff^40^. All these factors contribute to increased violence in the governmental health sector in Egypt compared with the private sector.

As mentioned by study respondents, more than two-thirds of the physical violence occurred during the night shifts, followed by evening shifts, which is in agreement with Abdel-Salam^5^. The increased physical violence in night and evening shifts could be attributed to the relative shortage of medical staff and security personnel. Also, night and evening shifts are often covered by junior physicians with limited experience in the absence of seniors, which increases liability for medical errors.

Regarding violent perpetrators, the current study revealed that patients' relatives were responsible for more than three-quarters of the violent events against physicians, which coincides with the results of previous Egyptian studies^23,25^. In Egyptian society, solid social relationships usually motivate family members and friends to attend to their patients^41^. Thus, the patient's relatives and friends might break their anger through violence toward medical staff.

A tiny fraction of the participating physicians pointed to the patients as violent perpetrators, which could be explained by the inability of patients incapacitated with their illness to exert physical aggression.

Nearly three-quarters of the participating physicians claimed that violence perpetrators were of low educational standards. In addition, almost half of all the respondents attributed hostile behaviors to the poor communication skills of the offenders. The association between violence toward healthcare providers and limited communication was proved by Ghiasee and Sağsan^42^.

The participating physicians stated that outbreaks of violence were triggered by a shortage of medical equipment/supplies, worrying regarding the patient's condition, and long waits to receive care, as mentioned by 49.5%, 45.7%, and 42.9% of participants, respectively. It is worth noting that the Egyptian healthcare sector services in a country with more than 100 million population in 2023,^26,27^which explains the overloaded healthcare system with a relative shortage of services. Additionally, the continuous emigration of Egyptian physicians to more developed countries further contributes to patients-physicians mis-proportion^43^.

Regarding the instruments used in aggression, three-quarters of the respondents declared that the offenders used their bodies to attack the physicians, whereas 29.5% of participants mentioned that blunt instruments were used. Most seriously, 7.6% and 1.9% of the participants were attacked by sharp and firearm weapons, respectively, which might reflect intense aggression and the associated grave risk.

The commonly reported attacks were pushing, pulling, throwing objects and fists. The traumas were mainly directed toward accessible areas in the upper half of the body. Similarly, Salem et al. 2022 reported that physicians’ heads, chests, and upper limbs were the most typical target areas for traumas^30^. Most seriously, some participating physicians reported attempts of stabbing and slashing with sharp weapons.

Regarding the physical consequences of the violence, three-quarters of participants experienced pain. Whereas simple wounds, including contusions, abrasions, and cut wounds, were mentioned by 22.9%, 16.2%, and 1.9% of participants, respectively. Most seriously, 4.8%, 3.8%, 2.9%, and 1.9% of violent attacks result in serious injuries, including firearm injuries, internal organs injuries, fractures, and burns. The previously reported physical outcome of violence toward Egyptian physicians during the COVID-19 crisis was much milder than that described in the current study. Salem et al. 2022 said that 4.5% of physicians had abrasions or contusions, and 0.6% of physicians reported simple wounds, whereas no serious injuries were reported^30^.

Complete healing is the usual outcome of violence-induced injuries, as reported by the current study participants. Nevertheless, a small fraction of them reported residual scars or even infirmities as a consequence of violence.

Regarding the immediate responses of attacked physicians, only 59% of them asked the security personnel for help, and only 34.3% called the police to handle the violent events. Similarly, in Egypt, Abdellah and Salama and Salem et al. mentioned that 76.2% of emergency medical staff and 75% of physicians did not report their exposure to violence^6,30^. Also, in Iraq, Lafta et al. declared that 69% of physicians did not report violence to the police^44^.

Considering the legal outcome of violence, only 14.3% of the physicians took legal and judicial actions. In contrast, no legal steps were taken by more than half of physicians in response to physical violence. Also, many physicians forgave the attackers and did not proceed with litigation procedures. The reluctance of physicians in this context could be attributed to dissatisfaction with legal responses to previous notifications and a lack of trust that appropriate action will be conducted^30,44^. Non-taking appropriate legal actions as a response to violence contributes to further violation of the rights of healthcare providers in the future.

The participating physicians believed that adopting rigorous legislation and strengthening the security issues are the most important preventive actions to protect medical staff. Also, participants recommended raising public awareness regarding the judicial penalties for violence against healthcare providers. In addition, it is necessary to improve medical service and decrease the workload by increasing the number of healthcare providers in Egypt.

### Strengths and limitations

The present study provided a comprehensive medicolegal analysis of physical violence toward physicians in Egypt. This research elucidates escalating and alarming violence toward Egyptian physicians, even after ending the COVID-19 crisis. The current results could govern future protective measures in the Egyptian healthcare sector. Also, the narrative review denoted that violence toward healthcare providers is a worldwide problem that was not adequately investigated from a medicolegal perspective.

The main limitation of the current study is a relatively small number of participants which could be attributed to the tendency of non-disclosure of work-related issues. Also, the narrative review of relevant studies has an inherited limitation of being less rigorous than a systematic review.

## Conclusion

Medicolegal analysis of attacks of physical violence reflected high aggression in healthcare institutes that were disproportionate to legal actions taken by physicians. The participating physicians reported a high frequency of physical violence and resultant physical injuries; some of these attacks involved using sharp and firearm weapons. Severe and life-threatening injuries were reported as well. In addition, some physicians suffered from lifelong scars or even permanent infirmities.

In the view of the current study, there is a necessity to adopt and implement strict laws to protect healthcare providers against violence. Also, the physicians should be encouraged to proceed with appropriate legal actions to prevent further violence in the future. Medicolegal aspects of violence against healthcare providers have gained limited attention; thus, conducting similar studies in different countries is recommended.

### Supplementary Information

Supplementary Table 1.

Supplementary Table 2.

Supplementary Information 3.

## Data Availability

The datasets used during the current study are available from the corresponding author upon reasonable request.
