# Supplementary material for: Medicolegal analysis of physical violence toward physicians in Egypt
Source: Sci Rep. 2024 May 13;14:10911. doi: 10.1038/s41598-024-60857-2 (PMC11091219; doi:10.1038/s41598-024-60857-2)
Supplement: Supplementary file 1 — Supplementary Table 1. [file 41598_2024_60857_MOESM1_ESM.docx]

**Supplementary Table (1): Relation between exposure to physical violence** **and other type of violence**

|  | **Did you experience physical violence in your medical practice?** | | | | **χ^2^** | **p** |
| --- | --- | --- | --- | --- | --- | --- |
|  | **Yes**  **(n = 105)** | | **No**  **(n = 145)** | |  |  |
|  | **No.** | **%** | **No.** | **%** |  |  |
|  |  |  |  |  |  |  |
| **Did you experience verbal violence in your medical practice?** | 104 | 99.0 | 116 | 80.0 | 20.924^*^ | <0.001^*^ |
| **Did you experience sexual harassment in your medical practice?** | 18 | 17.1 | 15 | 10.3 | 2.456 | 0.117 |

χ^2^: **Chi-square test**

p: p value for comparing between the studied categories

*: Statistically significant at p ≤ 0.05
