# Supplementary material for: Medicolegal analysis of physical violence toward physicians in Egypt
Source: Sci Rep. 2024 May 13;14:10911. doi: 10.1038/s41598-024-60857-2 (PMC11091219; doi:10.1038/s41598-024-60857-2)
Supplement: Supplementary file 2 — Supplementary Table 2. [file 41598_2024_60857_MOESM2_ESM.docx]

**Supplementary Table (2): Frequency of physical violence** **according to responses of participating physicians exposed to physical violence (n=105).**

| **Frequency of physical violence** | **No.** | **%** |
| --- | --- | --- |
|  |  |  |
| **Frequency of attacks of physical violence per year** |  |  |
| Once | 30 | 28.6 |
| Twice | 23 | 21.9 |
| Three times | 8 | 7.6 |
| Four times | 7 | 6.7 |
| Five times | 5 | 4.8 |
| >5 times | 32 | 30.5 |
|  |  |  |
| **Frequency of physical violence against physicians in Egypt in last years** |  |  |
| Increasing | 92 | 87.6 |
| Decreasing | 1 | 1.0 |
| Stationary | 12 | 11.4 |
|  |  |  |
| **Relation between COVID-19 and an increased frequency of physical violence** |  |  |
| Yes | 40 | 38.1 |
| No | 65 | 61.9 |
