# Supplementary material for: Medicolegal analysis of physical violence toward physicians in Egypt
Source: Sci Rep. 2024 May 13;14:10911. doi: 10.1038/s41598-024-60857-2 (PMC11091219; doi:10.1038/s41598-024-60857-2)
Supplement: Supplementary file 3 — Supplementary Information 3. [file 41598_2024_60857_MOESM3_ESM.docx]

The medical pages on social media that were used to distribute the questionnaire:

1. [**Egyptian Doctors**](https://www.facebook.com/groups/dwb.egypt/)

<https://www.facebook.com/groups/dwb.egypt>

1. [**Egypt Doctors**](https://www.facebook.com/groups/150127538680109/)

<https://www.facebook.com/groups/150127538680109>

1. [**Healthcare Providers Egypt - هيلثكير بروڤيدرز مصر**](https://www.facebook.com/groups/219971652540386/)

[**https://www.facebook.com/groups/219971652540386**](https://www.facebook.com/groups/219971652540386)

1. [**Medicine Society**](https://www.facebook.com/groups/1295691184321006/)

**https://www.facebook.com/groups/1295691184321006**
